# Toward sustainability and resilience in Chilean cities: Lessons and recommendations for air, water, and soil issues

**DOI:** 10.1016/j.heliyon.2023.e18191

**Published:** 2023-07-12

**Authors:** François Simon, Jorge Gironás, Javier Rivera, Alejandra Vega, Guillermo Arce, María Molinos-Senante, Héctor Jorquera, Gilles Flamant, Waldo Bustamante, Margarita Greene, Ignacio Vargas, Francisco Suárez, Pablo Pastén, Sandra Cortés

**Affiliations:** a Centro de Desarrollo Urbano Sustentable (CEDEUS), El Comendador 1916, Providencia, Santiago, Chile; b Departamento de Ingeniería Hidráulica y Ambiental, Pontificia Universidad Católica de Chile, Avenida Vicuña Mackenna 4860, Macul, Santiago, Chile; c Centro de Investigación para la Gestión Integrada del Riesgo de Desastres (CIGIDEN), Avenida Vicuña Mackenna 4860, Macul, Santiago, Chile; d Centro Interdisciplinario de Cambio Global, Pontificia Universidad Católica de Chile, Avenida Vicuña Mackenna 4860, Macul, Santiago, Chile; e Departamento de Ingeniería Química y Bioprocesos, Pontificia Universidad Católica de Chile, Avenida Vicuña Mackenna 4860, Macul, Santiago, Chile; f Escuela de Arquitectura, Pontificia Universidad Católica de Chile, El Comendador 1916, Providencia, Santiago, Chile; g Centro de Excelencia en Geotermia de los Andes (CEGA), Plaza Ercilla 803, Santiago, Chile; h Departamento de Salud Pública, Pontificia Universidad Católica de Chile, Diagonal Paraguay 362, Santiago, Chile; i Centro Avanzado de Enfermedades Crónicas (ACCDiS), Sergio Livingstone 1007, Independencia, Santiago, Chile

**Keywords:** Sustainability, Resilience, Cities, Air pollution, Water management, Soil quality, Energy efficiency, Chile

## Abstract

Achieving sustainability and resilience depends on the conciliation of environmental, social, and economic issues integrated into a long-term perspective to ensure communities flourish. Many nations are transitioning toward both objectives, while at the same time addressing structural concerns that have not allowed them to look after the environment in the past. Chile is one of these nations dealing with such challenges within a particular administrative context, an increasing environmental awareness, and a set of unique and complex geophysical boundaries that impose a plethora of hazards for cities, ecosystems, and human health. This paper presents recent accomplishments and gaps, mostly from an environmental perspective, on issues related to air pollution, the urban water cycle, and soil contamination, in the path being followed by Chile toward urban sustainability and resilience. The focus is on the bonds between cities and their geophysical context, as well as the relationships between environmental issues, the built environment, and public health. The description and diagnosis are illustrated using two cities as case studies, Temuco and Copiapó, whose socioeconomic, geographical, and environmental attributes differ considerably. Particulate matter pollution produced by the residential sector, drinking water availability, wastewater treatment, stormwater management, and soil contamination from the mining industry are discussed for these cities. Overall, the case studies highlight how tackling these issues requires coordinated actions in multiple areas, including regulatory, information, and financial incentive measures. Finally, the policy analysis discusses frameworks and opportunities for Chilean cities, which may be of interest when conceiving transitional paths toward sustainability and resilience for other cities elsewhere.

## 1 Introduction

According to the latest report of the Intergovernmental Panel on Climate Change [1], sustainability and resilience have become crucial in today’s era, especially in the context of global change (e.g. temperature rise and climate change, biodiversity loss, environmental pollution, migration from rural to urban areas). For instance, sustainability practices such as improving energy efficiency, transitioning to renewable energy sources, and adopting low-carbon technologies are key to mitigating greenhouse gas emissions and addressing climate change [2]. Promoting sustainable land use, protecting habitats, and adopting sustainable practices in agriculture, forestry, fishery, and mining activities can minimize the destruction of ecosystems and help to maintain biodiversity [1]. Sustainability efforts in urban areas can help to reduce pollution [3], energy consumption [4], and waste generation [5]. Resilience focuses on adapting to the impacts of climate change, such as temperature rise and extreme weather events, and rising sea levels [1]. Resilience also involves restoring degraded ecosystems, implementing conservation measures, implementing disaster preparedness plans, promoting climate resilient agriculture and water management systems, ensuring social inclusivity, and promoting community engagement in decision-making processes, among others [1].

Sustainability means addressing the challenges the population is facing, ensuring social and economic stability, quality of life, as well as the conservation of natural resources, ecosystems, and the environment [6]. Sustainability is not, and should not be, conceived as a single concept or a consistent set of concepts, but as an approach of community-based thinking in which the environmental, social, and economic issues are integrated with a long-term perspective [7]. Resilience is another approach aiming to accomplish maximum good for societies and the environment across various fields and focus areas, including engineering, energy systems, land-use, and urban planning, among others [8]. The approach of resilience theory resides in strengthening the adaptive capacity of interconnected social, economic, and ecological systems to cope with a hazardous event, trend, or disturbance, responding or reorganizing in ways that maintain their essential function, identity, and structure [1].

Resilience thinking involves fundamental assumptions that may differ and even contradict those of the sustainable approach [9]. For instance, a system could be sustainable but not resilient, as sustainability rather focuses on ensuring that the limits of the global system are not breached when meeting socio-economic demands, while resilience emphasizes maintaining systems’ functionality. Also, it should be noted that although both concepts are normative, there are more ambiguities regarding resilience [10]. The literature is dominated by three generalized management frameworks for organizing sustainability and resilience [11]: (1) sustainability as a component of resilience, (2) resilience as a component of sustainability, and (3) sustainability and resilience as separate objectives. According to Marchese et al. [11], the best examples of these frameworks’ implementations are built on minimizing conflicts between sustainability and resilience. Indeed, resilience thinking may provide valuable insights into sustainability and vice versa.

Developing nations of the global South are facing the challenge of adapting approaches in which sustainability and resilience coexist, while at the same time typically dealing with problems such as the absence of strategic economic vision, lack of coordination between different levels of government and institutions, compartmentalized sector-based policy-making, and ineffective policy interventions [12]. The primary concern in many of these societies has often been to earn a living, build communities and cities considering cost-effective options, mainly those exploiting fossil fuel sources to cope with the increased demand for energy, and it is only recently that they are learning how to look after the environment [13]. In Chile, fast economic growth and rapid population increase have led to unprecedented urbanization that has transformed the country’s population distribution from 58% urban in 1950 to more than 83% urban in just four decades. In 2023, Chile counts 44 urban agglomerations of over 50,000 inhabitants, and 49% of the total population lives in the four largest urban agglomerations of Santiago, Valparaiso, Concepción, and La Serena [14], while urbanization is expected to reach 92% by 2050 [15]. Although urban and industrial areas cover 0.7% of the country’s land surface [16], they are responsible for the vast majority of energy-related carbon emissions [17]. As cities continue to grow, urban sustainability and resilience have become important goals, particularly in the face of global warming and climate change, as means to reduce environmental, socioeconomic, and political uncertainty [8].

Chile is a country transitioning toward the challenges of sustainability and resilience within a context defined by several attributes linked to its landscape. A variety of different geo-climatic settings across latitudes and longitudes take place, with strong gradients of climate variables, a diversity of geomorphologic and geochemical driving forces, and the presence of large geophysical features including the Andes, the Pacific Ocean, and the Atacama Desert in the north [[18], [19], [20], [21]]. Despite its strong natural variability, administrative decision-making in Chile is centralized, with regional offices and agencies of centralized institutions having more power than local governments [22]. In addition, Chile signed up in 2010 as the first South American member of the Organization for Economic Co-operation and Development (OECD), and thus economic aspects are critical to the central government [23]. Furthermore, increasing environmental awareness is taking place in Chile [24]. This increased public perception started in the year 2008, with the release of the Environmental Impact Statement for the Hydro Aysen power plant project in Patagonia, which was finally withdrawn due to the social pressure supported by political, technical, and public opposition [[25], [26], [27]]. Subsequently, the community has opposed the implementation of energy and mining projects based on their anticipated environmental and social impacts [[28], [29], [30]]. Other recent examples of advances toward more environmentally friendly development in the country include the phasing-off of plastic bags in food and retail stores [31], a remarkable transition of the energy matrix to a higher share of renewable energies [32], the willingness to pay for greener sources of energy among Chilean citizens [33], the taxation scheme for CO_2_ emissions [34,35], the law for the protection of urban wetlands [36], and the national framework law of climate change [37].

At the same time, the country faces socioeconomic fragilities and a lack of critical infrastructure to cope with a plethora of natural hazards, such as earthquakes and tsunamis, floods, droughts, landslides, volcanic eruptions, large-scale fires, extreme turbidity events, among others [[38], [39], [40], [41], [42], [43]]. High levels of exposure and vulnerability in many cases have led to increased risks, particularly in urban environments [44]. The severity and frequency of natural events have motivated a clear focus on resilience throughout the history of the country [45], although *ad hoc* response to emergencies has been favored over planning. Indeed, resilience in livelihoods should be fundamental in the vision of sustainability from societies, particularly the Chilean one. Chile has been successful in controlling the vulnerability to earthquakes but has a much lower level of success for other natural hazards. For example, poor land-use planning is considered a major issue, since it has led to increased levels of risk exposure, particularly in areas of low-income and vulnerable communities [46]. Recently, Chile has launched several initiatives to increase resilience through a more comprehensive/holistic approach, in which territorial and urban planning are considered central elements to cope with natural hazards [41]. Examples of these initiatives are the variety of risk management studies developed by municipalities and regions after the 2010 earthquake and tsunami [47], the release of a guide for natural risk analysis in land planning [48], the creation of national research centers dedicated to studying and managing risks (i.e. CIGIDEN, CITRID, CR2, CEDEUS) [49], the creation of a government commission aiming to elaborate a policy report to guide the development of top world scientific and entrepreneurial expertise to deal with natural hazards [50], the national policy for the management of disaster risk [51], and the law 21364 that creates the national system of prevention and response against natural disasters [52].

The objective of this paper is to provide recommendations for more sustainable and resilient cities in Chile. For that purpose, we discuss a series of solutions that are required to be implemented on multiple scales and areas, including assessment and information, regulation and financial incentive measures, and infrastructure and technologies. In particular, we focus on recent accomplishments and gaps -as well as future challenges for sustainability and resilience in Chile-for tackling environmental issues related to (1) airborne particulate matter (PM) pollution produced by the residential sector (excluding other stationary and mobile sources), (2) availability and distribution of drinking water, wastewater treatment, and stormwater management, and (3) soil contamination from the mining industry, and their relationship with public health and the built environment within cities. The outline of the paper is as follows. Section 2 presents a literature review of the most critical environmental challenges related to air pollution, the urban water cycle, and soil contamination at a broad national level. Section 3 illustrates these issues with two case studies in two Chilean cities. Section 4 presents a discussion about the policy frameworks in force, as well as opportunities for the application of new strategies and policies. Finally, the main conclusions and recommendations for more sustainable and resilient cities are provided in section 5.

## 2 Materials: a literature review of the challenges for sustainability and resilience in Chile

**Fig. 1:**
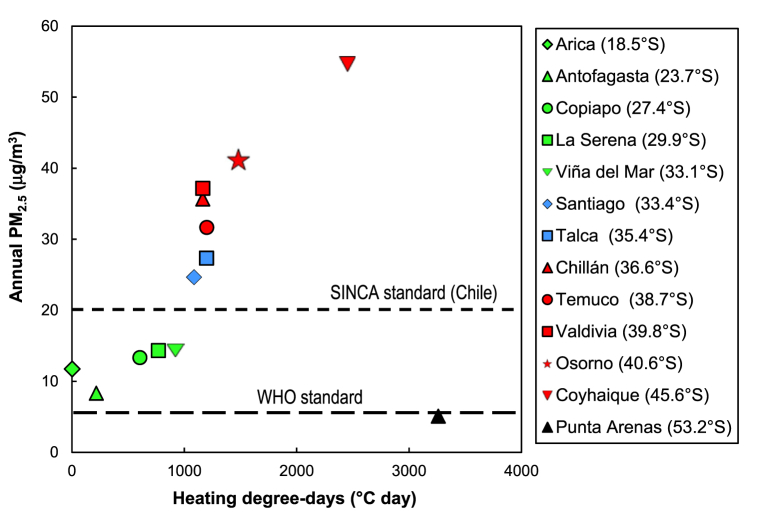
Annual ambient PM_2.5_ concentrations (averaged for 2016–2018) in Chilean cities plotted against heating degree days. Horizontal lines show national [121] and international [122] air quality standards. Adapted from Villalobos et al. [55].

**Fig. 2:**
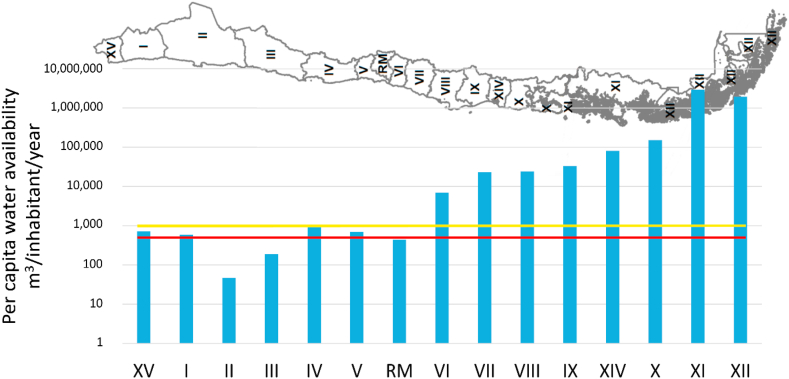
Water availability in Chilean regions (runoff). Horizontal lines represent threshold values for water scarcity (<1,000m^3^/inhabitant/year) (yellow line) and absolute water stress (<500m^3^/inhabitant/year) (red line). Elaborated using data from DGA [83].

**Fig. 3:**
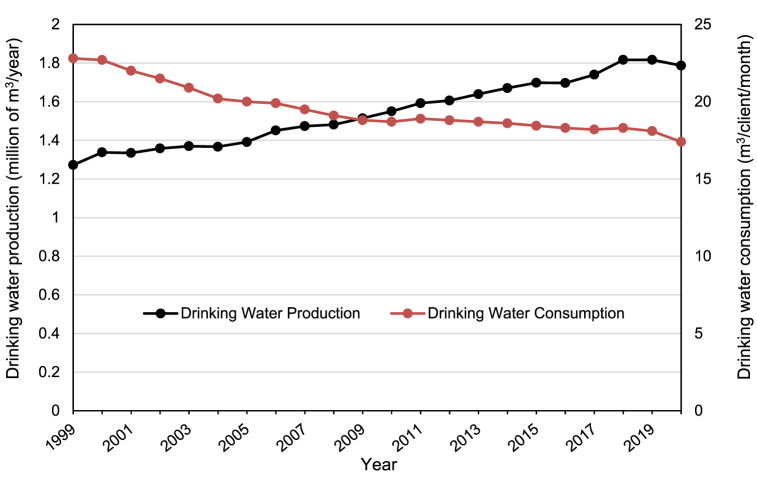
Water production and consumption in Chile (1999–2020). Data were obtained from SISS [86,96].

Sustainability and resilience largely depend on the status of critical resources (e.g., air, water, and land/soil) and the processes that utilize and/or impact them. Despite the variability in climatological, hydrological, geochemical, economic, and social conditions of Chilean cities, some national trends can be identified regarding the actual status and challenges associated with each one of these environmental matrices and urban development. This section reports the most relevant challenges associated with ambient particulate matter (PM)-related air pollution, urban water cycle, and soil quality (metal-metalloids presence) for Chile at a broad national level. For that purpose, we use a pithy approach to compile information from the literature in the form of tables summarizing air-, water-, and soil-related challenges (Table 1 to Table 3). Table 1 presents the impacts, causes, and current action plans associated with particulate matter (PM)-related air pollution. Table 2 and Table 3 introduce the impacts, causes, and current action plans associated with the urban water cycle and soil quality (metal-metalloids presence), respectively. Furthermore, some points from these tables are illustrated in Fig. 1, Fig. 2, Fig. 3. Fig. 1 shows that all the largest urban areas across the country have an annual mean PM_2.5_ concentration above the World Health Organization (WHO)standard with higher annual concentrations in locations with higher heating demand. Fig. 2 displays the water stress level of the Chilean regions and highlights the low runoff water availability of northern regions. Fig. 3 presents the trends of national drinking water production and consumption, showing that between 1999 and 2020, drinking water production increased by more than 40% (i.e. from 1.27 to 1.79 million m^3^/year), while the consumption has decreased by 24% (i.e. from 22.8 to 17.4 m^3^/month).

**Table 1:** Particulate matter (PM)-related air pollution.

	
Impacts	- More than 10 million Chileans (53% of the population) are exposed to ambient PM_2.5_ concentrations above the national air quality standard [53]. - Around 3640 cases of premature mortality due to air pollution-related cardiovascular and respiratory diseases were estimated in 2018 [54]. - Seven south-central cities have ambient annual mean PM_2.5_ concentration above the national air quality standard (Fig. 1) [55]. - High PM_2.5_ concentrations are also present in indoor environments [56]. - Evidence of positive relationships between ambient PM levels and the rates of mortality and morbidity for cardiovascular and respiratory causes [57]. - Greater risk of mortality and morbidity for cardiovascular and respiratory diseases when people are exposed to air polluted with wood smoke [57].
**Causes**	- Significant demand for space heating energy in central-south Chile [58] due to the cold and humid climate [59]. - Wood biomass is the main energy fuel for residential space heating and cooking in south-central Chile [56]. - Wood biomass burning is the main source of PM_2.5_ in cities of central-south Chile [54,57]. - Wood is the most economical and accessible source of energy [60]; Large use of cheaper wet wood [60]. - Use of open fireplaces and old wood stoves with low thermal efficiency [61,62]. - Low energy performance of housing [[63], [64], [65]]: 80% of dwellings have little or no thermal insulation [61].
**Current plans for action**	- Building thermal regulation standards: Article N°4.1.10 of the General Law of Urban Planning and Construction (OGUC) [66]. - Air Quality Management Plans (PPDA) applying to highly polluted cities (south of Santiago): provide grants for house thermal retrofit and space heating system upgrade. Retrofits under PPDA must comply with higher limiting standards for insulating fabric elements than in the OGUC [67]. - Program for the Regeneration of Social Housing Complexes (PRCH) of the Supreme Decree Nº18 of 2017: include state subsidies for building thermal retrofit, which must comply with higher limiting standards for insulating fabric elements than in the OGUC [68,69].

**Table 2:** Urban water cycle.

		
Availability and quality	Impacts	- Water scarcity: almost 65% of the population has access to less than 1,000 m^3^/inhabitant/year of water (Fig. 2) [83]. - Discharge volumes in the Maipo Basin (the principal supplier of potable water for the Santiago metropolitan area) are estimated to decrease by up to 40% in the future [84]. - Droughts impact water quality [85,86]. - High turbidity events impact the operation of the drinking water supply systems [86,87]. - High concentrations of metals and metalloids in rivers of northern-central zones [88] and groundwaters [21].
	Causes	- Climate change: lack of precipitation and drought [89]. - Intensive use of water by mining and agriculture activities [90]. - Climate change: warm storms in the Maipo River [91] causing high turbidity events. - Geogenic metal enrichments in addition to anthropogenic activities in the northern and central zones of Chile [92].
	Current plans for action	- National Strategic Plan for Disaster Risk Reduction in the Water Sector 2020–2030 [93]. - Framework law of climate change: establish water balance and projections, information on quantity, quality, infrastructure, and institutions that intervene in the decision-making process regarding water resources and propose a set of actions to safeguard water security [37]. - Construction of reservoirs to increase the autonomy of water production systems and reduce the impact of turbidity events [94]. - Expansion of the monitoring network, promotion of scientific research, and early warning systems [94]. - More comprehensive surveillance programs for ambient water quality standards [21]. - Establishment of regulations and standards for the collection, reuse, and disposal of greywater [95].
**Distribution and consumption of drinking water**	**Impacts**	- Total urban water production increased by 40% between 1999 and 2020, up to 1,787 million m^3^ per year (Fig. 3) [86,96]. - There is a national average of 33.4% of non-revenue water in 2020 [86]. - Water leakages in the distribution system are estimated to account for 74% of non-revenue water [97]. - Access to drinking water, sewage, and wastewater treatment in urban areas in 2020 reached 99.9%, 97.3%, and 97.3% [86]. - Water consumption per capita in Chile: 17.4 m^3^/client/month in 2020 (i.e. approximately 161 L/day/person) (Fig. 3) [86].
	**Causes**	- Urban population growth [98]. - Inefficient water markets due to lack of complete information, poorly flexible distribution systems, high transaction costs [99], and fragmented governance of water resources [100].
	**Current plans for action**	- Water leakage control strategies: pressure management [101], night flow analysis in individual District Metering Areas (DMAs) [102]. - Incorporate network monitoring systems and increase the replacement of obsolete infrastructure [103].
**Wastewater treatment**	**Impacts**	- Alteration of marine ecosystem functioning: effects on the biochemical composition of sediments, production of organic enrichments, which later degradation lead to lower oxygen concentrations and hypoxia [104]. - Pharmaceuticals and personal care products found in effluents from wastewater treatment plants involve endocrine disruption effects on freshwater fish [105]. - Highly energy-intensive activated sludge technology with important volumes of sewage sludge [106,107].
	**Causes**	- Excessive discharge of nutrients (nitrogen and phosphorus) to water bodies [21]. - Marine outfalls collect 11% of wastewaters: 33 of the 301 treatment systems include neither biological nor chemical processes [86]. - Tertiary treatment is not performed for nutrient removal in wastewater treatment plants [21]. - The lack of secondary environmental standards (i.e. regulating ambient pollutant concentration) in Chilean water bodies, similar to Total Maximum Daily Loads (TMDL) [108]. - Plants are not designed to remove pollutants such as pharmaceuticals and personal care products [105]. - Use of energy-intensive technology in various stages of the treatment process [109,110], in an urban water cycle not optimized in terms of energy efficiency [111].
	**Current plans for action**	- Development of secondary environmental standards: Five of the 101 watersheds have secondary environmental standards [112]. - Modification of the Supreme Decree Nº90 standards for liquid waste discharges into marine and continental surface waters [113]. - Define watershed-specific ambient water quality standards and more stringent discharge standards for treated wastewaters [21].
**Stormwater management**	**Impacts**	- Large stormwater volumes and discharges cause urban flooding and fluvial ecosystem deterioration [114,115]. - Degradation of the quality of water bodies receiving stormwaters [116].
	**Causes**	- The replacement of the vegetation cover with impervious surfaces [117]. - The occupation and poor management of floodplains [118]. - Urban areas as nonpoint sources of metals, oxygen-demanding substances, and suspended solids transported in stormwaters [116].
	**Current plans for action**	- The urban drainage manual provides planning strategies, designing tools, and standards for stormwater control (i.e. conveyance, infiltration, and storage) [119]. - The sustainable urban drainage guide for southern Chile provides information about natural drainage systems, including green infrastructures, the national regulatory framework, and planning strategies for their implementation [120].

**Table 3:** Soil quality and urban planning.

	
Impacts	- Accumulation of toxic metal-rich wastes (i.e. tailings) from mining activities in urban and peri-urban areas [70,71]. - Dispersion of toxic metals from mine tailings without proper safe closure measures [72]. - Mining accounted for ∼10% of the total GDP in 2018 (over 30% in some regions, up to 53% in the Antofagasta region) [73]. - Natural and anthropogenic soil enrichment with toxic metals and metalloids [70,74,75]. - Mine tailings: 85% of 764 tailings in Chile are either inactive or abandoned [76].
**Causes**	- Absence of regulations establishing substance concentration threshold values by soil use, as in Canada [77] and Brazil [78]. - Urban planning legislation does not consider soil quality and health impacts of mine tailings [70]. - Urban planning legislation does not explicitly consider public exposure to metals in soil risk assessments [79]. - Local background not defined for the establishment of soil quality guidelines [70] - Urban planning legislation is vague in the definition of “risk areas” (i.e. Article N°2.1.17) [66].
**Current plans for action**	- Polymetals Health Initiative of the Arica and Parinacota region as a local policy [80]. - Guide for the management of soils with the potential presence of pollutants [81]. - New soil bill aiming at protecting and restoring soils. Limiting urban development in contaminated areas is currently being discussed [82].

## 3 Case studies: Temuco and Copiapó

**Fig. 4:**
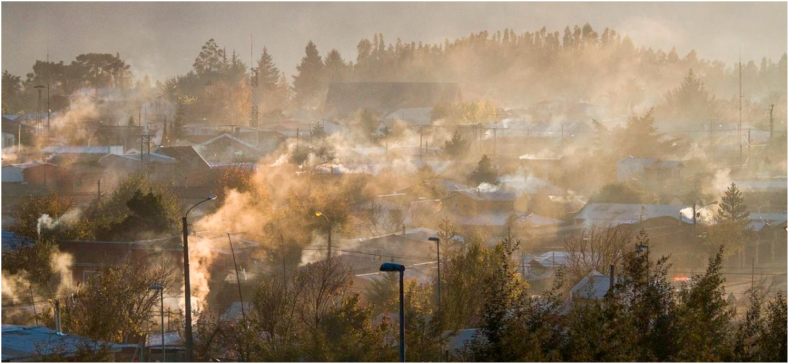
Wood smoke air pollution in Temuco, June 2020. David Cortes Serey/AgenciaUno [130].

**Fig. 5:**
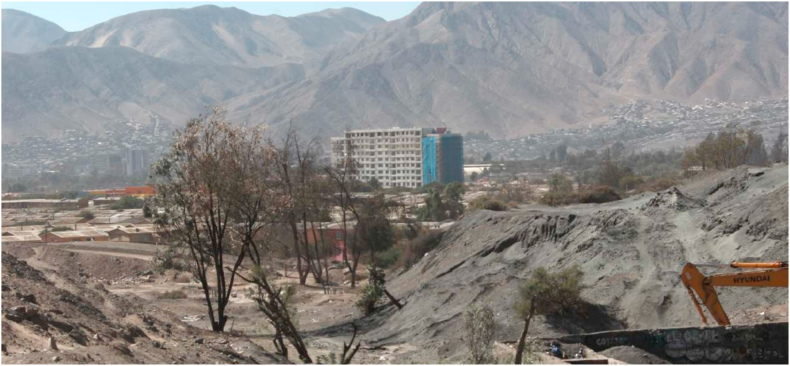
Abandoned mine tailing, sector El Palomar, Copiapó. April 2015. Raimundo Gómez [131].

**Fig. 6:**
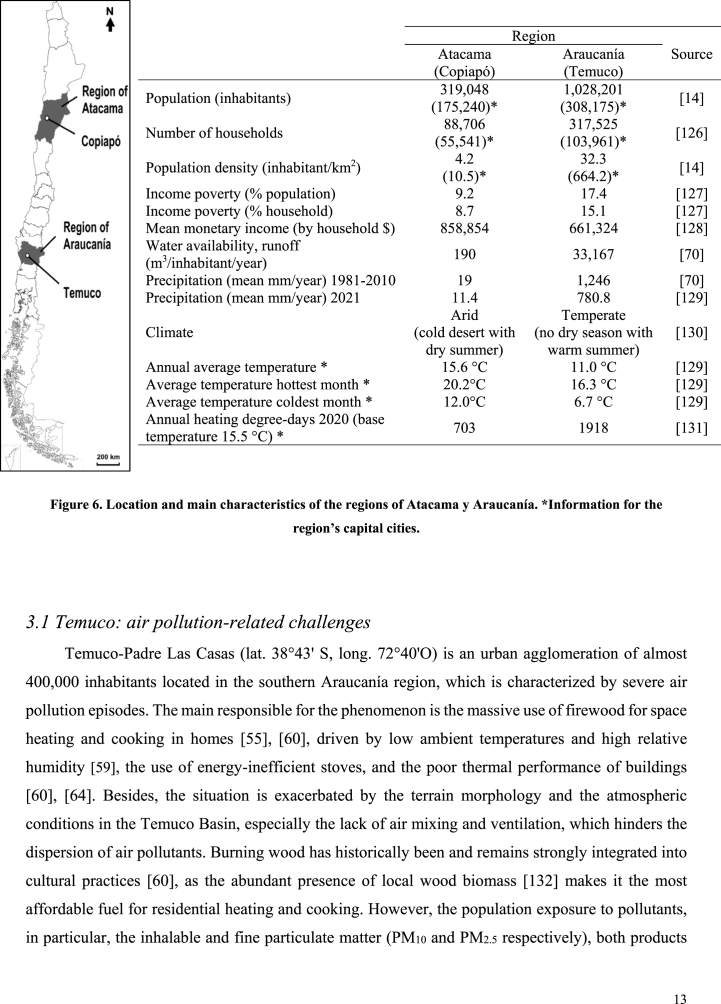
Location and main characteristics of the regions of Atacama y Araucanía. *Information for the region’s capital cities.

In this section, we illustrate some of the sustainability challenges previously identified using two case studies. The first one relates to the implications of residential wood-based energy consumption on air pollution in Temuco, the capital of the Araucanía region. Temuco (308.175 inhabitants) was selected as a case study because it is one of the most highly wood-smoke-polluted cities in the world [57] (Fig. 4). Like most urban areas in central-south Chile, Temuco experience episodes of severe air pollution during winter. The second case study presents the challenges associated with water scarcity, soil quality problems, and public health implications in the city of Copiapó, the capital of the Atacama region. Copiapó (175.240 inhabitants) was selected because it presents serious environmental issues related to water scarcity and soil pollution [70,123]. Like most urban areas of central-north Chile, Copiapó is characterized by mining activities, which are operating in nearby urban and peri-urban areas (Fig. 5). Fig. 6 shows the location of both regions and cities, complemented by relevant background information and characteristics. The Araucanía region has a larger and denser population, with lower average income, whereas Copiapó, in the Atacama Desert, is a region with significantly lower water availability.

**Table:** 

	Region		
	Atacama (Copiapó)	Araucanía (Temuco)	Source
Population (inhabitants)	319,048 (175,240)*	1,028,201 (308,175)*	[14]
Number of households	88,706 (55,541)*	317,525 (103,961)*	[124]
Population density (inhabitant/km^2^)	4.2 (10.5)*	32.3 (664.2)*	[14]
Income poverty (% population)	9.2	17.4	[125]
Income poverty (% household)	8.7	15.1	[125]
Mean monetary income (by household $)	858,854	661,324	[126]
Water availability, runoff (m^3^/inhabitant/year)	190	33,167	[83]
Precipitation (mean mm/year) 1981–2010	19	1,246	[83]
Precipitation (mean mm/year) 2021	11.4	780.8	[127]
Climate	Arid (cold desert with dry summer)	Temperate (no dry season with warm summer)	[128]
Annual average temperature *	15.6 °C	11.0 °C	[127]
Average temperature hottest month *	20.2 °C	16.3 °C	[127]
Average temperature coldest month *	12.0 °C	6.7 °C	[127]
Annual heating degree-days 2020 (base temperature 15.5 °C) *	703	1918	[129]

### 3.1 Temuco: air pollution-related challenges

**Fig. 7:**
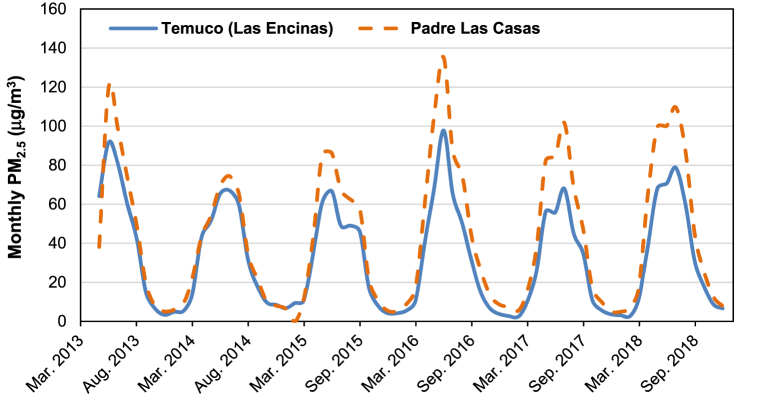
Evolution of monthly ambient 24-h PM_2.5_ measured in Temuco and Padre Las Casas monitoring stations, from March 2013 to December 2018. Data obtained from http://sinca.mma.gob.cl.

**Fig. 8:**
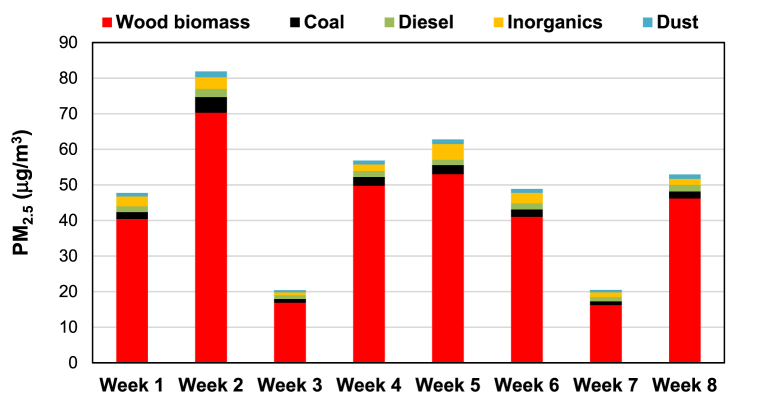
Distribution of PM_2.5_ sources in Temuco for the 2014 winter season (from July 15th to September 13th). Modified from Villalobos et al. [55].

Temuco-Padre Las Casas (lat. 38°43′ S, long. 72°40′O) is an urban agglomeration of almost 400,000 inhabitants located in the southern Araucanía region, which is characterized by severe air pollution episodes. The main responsible for the phenomenon is the massive use of firewood for space heating and cooking in homes [55,60], driven by low ambient temperatures and high relative humidity [59], the use of energy-inefficient stoves, and the poor thermal performance of buildings [60,64]. Besides, the situation is exacerbated by the terrain morphology and the atmospheric conditions in the Temuco Basin, especially the lack of air mixing and ventilation, which hinders the dispersion of air pollutants. Burning wood has historically been and remains strongly integrated into cultural practices [60], as the abundant presence of local wood biomass [132] makes it the most affordable fuel for residential heating and cooking. However, the population exposure to pollutants, in particular, the inhalable and fine particulate matter (PM_10_ and PM_2.5_ respectively), both products of the combustion of wood biomass, suggests a greater risk of mortality and morbidity for cardiovascular and respiratory diseases [57]. The Temuco-Padre Las Casas agglomeration is regularly declared saturated due to PM_2.5_ concentrations exceeding daily and annual standards. Concentrations of PM_10_ and PM_2.5_ can reach values of over 600 μg/m^3^ during certain hours of winter days [133]. Fig. 7 shows the evolution of monthly ambient PM_2.5_ measured at two monitoring sites in the agglomeration since 2013. Annual averages are well above the WHO guideline of 5 μg/m^3^ [122] and exceed Chile's national ambient air quality standard of 20 μg/m^3^ [121]. Ambient PM_2.5_ varies seasonally, with higher values in the winter season, when 85% of PM_2.5_ comes from wood biomass burning (Fig. 8) [55].

**Fig. 9:**
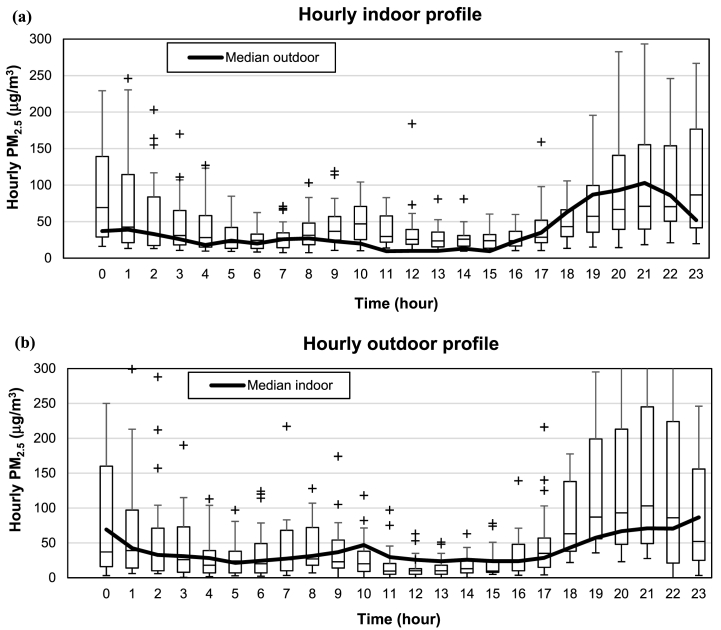
Comparison of hourly indoor (a) and outdoor (b) PM_2.5_ measurements for 25 households in Temuco. Modified from Jorquera et al. [56].

For indoor environments, the situation is equally concerning. A field measurement campaign at 63 households conducted during the winter of 2014 in the Temuco urban agglomeration provided the first set of indoor air quality measurements, as well as the first empirical evidence of the source of PM_2.5_ [56]. In this study, measurements indicate that indoor PM_2.5_ concentrations tend to balance rapidly with those from outside, but the indoor median PM_2.5_ concentration remains on average 10% higher than the outdoor median value. Using a mass balance approach, Jorquera et al. [56] show that 68% of indoor PM_2.5_ comes from outdoor infiltration, highlighting poor housing airtightness. Fig. 9, which compares hourly boxplots of indoor and outdoor PM_2.5_ concentrations, shows that median indoor concentrations (Fig. 9a) are higher than the median outdoor ones (Fig. 9b), except between 4 p.m. and 10 p.m. when wood-fueled cooking and space heating equipment is turned on.

Wood burning pollution is an issue in central-south Chile, which can severely affect the population’s health. Better air quality must be ensured by reducing the use of wood as the main energy source for space heating and cooking. To do so, fast implementation and effective short-term solutions include the improvement of the building envelope (i.e. thermal insulation and air tightness) and the replacement of old stoves with more efficient ones. Wood burning with optimal air inlet conditions may generate up to 10 times fewer pollutant emissions than operating with low air intake [[134], [135], [136]]. Between 2011 and 2021, government incentives led to changing of almost 12,800 wood stoves in Temuco [137], while subsidies for thermal insulation retrofits (started in 2015) reached 16,700 households by October 2020 [138]. Additionally, in May 2019, the regional office of the Ministry of the Environment allocated private consultancy tenders to evaluate the impact of district heating systems on PM emissions for new building blocks in the Temuco-Padre Las Casas urban area [139]. However, while the application of such policy measures is generally limited to the main urban areas, where Air Quality Management Plans (PPDA in Spanish) are in force, there is a lack of mitigation plans in rural sectors, as small towns nearby Temuco, like Angol, Victoria, Villarrica or Traiguén also present high levels of air pollution due to residential wood burning.

### 3.2 Copiapó: water- and soil-related challenges

Copiapó (lat. 27°22′S, long. 70°19′O) is an example of a city facing several water-related challenges. Located in the Atacama Desert in northern Chile, one of the driest places in the world, Copiapó has an average annual precipitation of only 12 mm. The Copiapó River is insufficient to meet the demands of the urban, agriculture, and mining sectors, as it has an annual average flow of 0.61 m^3^/s measured at the flow gauge of La Puerta [140] and runs almost dry through the city due to upstream diversions. The major water stress situation, coupled with the lack of an effective management model, has led to intense competition for water resources, the over-granting of water rights of use (i.e. the volume of water rights allocated is larger than water availability), and the exploitation of groundwater resources [123,141].

The sustainability of the urban water cycle of Copiapó is affected by various crucial factors. First, non-revenue water due to leakages reached 33.1% in 2021 [86], and a significant effort should be made in the first place to improve distribution efficiency. Second, the increasing demand in the past decades from the urban, agricultural, and mining sectors in the region of Atacama, where Copiapó is located, has forced the exploitation of new aquifer sectors with deeper water table levels, and this has increased energy requirements for pumping. As a comparison, groundwater pumping alone in Copiapó is more energy demanding than abstraction, elevation, drinking water treatment, and wastewater treatment in Santiago, the capital of Chile with 8 million people (i.e. 1.22 kWh/m^3^ against 0.36 kWh/m^3^, respectively) [142]. Third, groundwater usually presents high levels of dissolved salts. Despite more intensive treatments, the quality of drinking water from ground sources has worsened [97], as treatments often struggle to meet the Chilean standards for nitrate, chloride, sulfate, and total dissolved solids [92]. To that concern, seawater desalination appears as a possible alternative for potable water production [143,144], and the first stage of a large seawater desalination plant was inaugurated in January 2022 in the region. In its final construction stage, the plant will have the capacity to produce 1.2 m^3^/s of standard-compliant drinking water, to cover the demand of approximately 210,000 people in the districts of Copiapó, Chañaral, Caldera, and Tierra Amarilla (i.e. 75% of the regional population), for a total electrical energy consumption of 2.8 kWh/m^3^ [145]. Annually, this equals 106 GWh, the equivalent total energy consumption of approximately 13,000 households, considering the national Chilean home average of 8,083 kWh/year [61]. This type of infrastructure project will undoubtedly increase the resilience to water scarcity in the region, but equally important is the integration of sustainable management strategies to water desalination projects, such as renewable electricity generation and effective environmental impact mitigation plans, to ensure long-term benefit. Indeed, apart from the extra pressure the plant may provoke on local electricity markets in the context of energy transition [60], other concerns remain regarding brine discharge to the marine environment, which may have adverse effects on seawater and sediment quality, impair marine life, and the functioning of coastal ecosystems [146,147].

Regarding wastewater treatment, the activated sludge technology used in Copiapó does not include nutrient removal. To protect water resources in this water-stressed area, it becomes mandatory to set total maximum daily loads for the entire Copiapó watershed and implement nutrient removal in wastewater treatment processes. Furthermore, wastewater recycling should be strongly considered to cover increasing water demands in urban areas, as it could also reduce the pressure on groundwater resources. To this concern, the National Congress approved Law 20.075 in 2018 [95] to legislate the collection, reuse, and disposal of greywater. However, the conditioning regulation that defines sanitary standards for greywater reuse, as required by Law 20.075, remains to be released by the Ministry of Health, so that water reuse schemes could legally be implemented. In Copiapó, a clearer legal framework defining the ownership of treated wastewater, the implementation of environmental policies on pollution control, the integration of reclaimed water in public policies, and government funding for water recycling and reuse projects are key to overcoming the most important barriers for the implementation of such schemes [148].

**Fig. 10:**
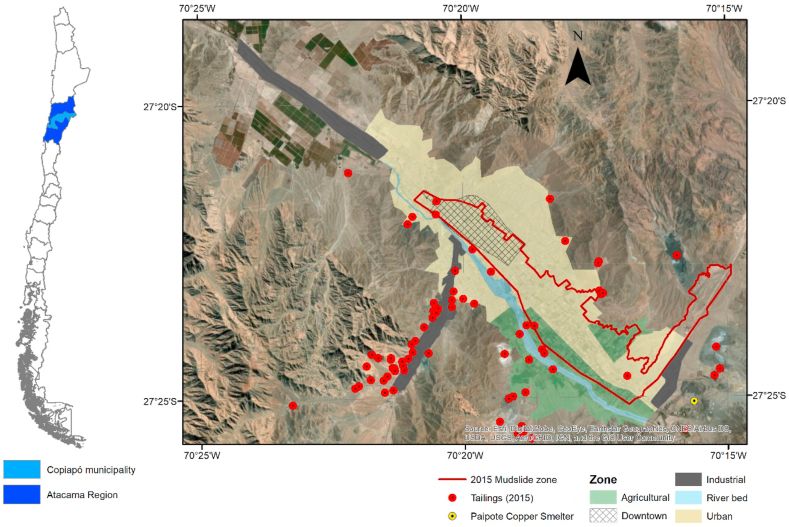
Areas of main tailings and landslides in the urban and peri-urban areas of Copiapó. Relevant land uses are also shown. Source: Own elaboration.

Copiapó is also a good example of soil-related problems. Its location in the Atacama Desert has led to a strong relationship with the mining industry since the foundation of the city in the 18th century, as the hyperaridity facilitated the accumulation of several elements in economically valuable quantities [149]. The urban development of Copiapó has been strongly linked to the development of the mining industry, beginning in the 19th century. Today, mining is the main economic activity in the region. Unfortunately, tailings are a negative externality of this activity that has historically affected Copiapó. Currently, there are 84 mine tailings in the municipality of Copiapó [76], with more than 50 of them being inside the city or within 2 km of the urban area, a consequence of poor urban planning and uncontrolled urban growth (Fig. 10). Furthermore, some of these mine tailings were exposed to the 2015 mudslide reported elsewhere [115,150]. Carkovic et al. [70] suggested that these tailings, along with a copper smelter, are a source of metals and metalloids in the city. They showed that copper (Cu) concentrations in soil and street dust samples from the urban area of Copiapó exceeded international guidelines for residential and industrial use soils. Some arsenic (As), zinc, and lead concentrations were also found to be above residential use guidelines. These concentrations were generally above the background or natural concentrations, indicating metal enrichment [151]. Furthermore, some elements such as As and Cu are in concentrations high enough to represent a potential risk to the population [152].

Intense rainfall events with consequences on productive activities, infrastructure, and human lives may occur in Copiapó. In March 2015 several flood events affected the Atacama region [153], including Copiapó [150]. The flood was caused by ∼45 mm of rainfall over the Copiapó River basin, the equivalent of two years of rainfall registered during just three days. Such conditions are expected every 40–60 years [150]. Vast parts of the city were flooded (i.e. 50% of the urban area), with flow depths reaching up to 2 m. Nine casualties and thirteen missing people were informed [154]. The flood also raised concerns about higher exposure to pollutants, but studies suggested that concentrations in the muds were within standard levels [155] and that the rainfall event cleaned street dusts [156]. Nevertheless, mud depositions later increase ambient PM_10_ concentration due to suspended road dust [157]. The hydraulic design of the Kaukari fluvial park performed well during the 2015 floods, as only the vegetation was lost but the built structures were not damaged [158]. This large-scale urban infrastructure, under construction since 2011, aims to restore the abandoned riverbed of the Copiapó River in its section through the city and provide more protection against floods. At the same time, the implementation of green spaces and wetlands within the park aims to bring social and environmental services [159]. By 2018, a third of the 160-ha park was completed [158].

The Copiapó case described here shows how urban growth without considering soil quality aspects may lead to potential environmental and public health risks, challenging resilient and sustainable urban development. A lack of more comprehensive information about environmental baselines on soil and water undermines efforts toward effective and efficient environmental guidelines. On the other hand, the fluvial park developed in the city is an example of actions toward increasing resilience while also improving the quality of life of the population.

## 4 Discussion: policy analysis

The case studies of Temuco and Copiapó reveal the different environmental challenges driven by the impacts of human settlements on distinct landscapes along the country's territory. This section presents policy frameworks in place, as well as opportunities for the application of new regulations for urban sustainability and resilience.

### 4.1 Solutions for heating-related air pollution: retrofitting existing homes

It is widely recognized that building energy retrofitting offers great opportunities to reduce energy consumption and emissions [[160], [161], [162], [163], [164]] and/or improve thermal comfort [[165], [166], [167]]. Also, retrofitting existing buildings is generally more environmentally friendly and economical than demolishing and rebuilding [[168], [169], [170], [171], [172]]. Common retrofit procedures generally involve first a building energy audit, then a performance assessment and diagnosis, and finally an economic analysis, to identify the areas of energy wastage and select the best retrofit options based on their cost-effectiveness potential [173,174]. Refurbishment interventions may include retrofitting the building fabric envelope with better thermal insulation and airtightness, replacing windows (i.e. multiple glazing, low emissivity coating, shading), implementing appropriate ventilation strategies (i.e. natural, mechanical, heat recovery) to control indoor air humidity and pollution, upgrading to low-energy equipment (i.e. energy-efficient lighting and appliances) and implementing renewable energy technologies (i.e. ground- or air-source heat pump, solar thermal and photovoltaic). However, since only the appropriate combination of these interventions can effectively improve the building energy performance, retrofitting processes must be properly designed to be cost-optimal. For this purpose, it is fundamental to adopt a comprehensive step-by-step approach aimed at facilitating the coordinated implementation of the different retrofit interventions [175].

Improving actual rates of building retrofits also requires the enforcement of various retrofit policy instruments [176]. These can be classified into four main categories: (1) Direction and command instruments must include setting overall retrofit strategies, targets, requirements, and guidelines, to assist stakeholders in better understanding the benefits of energy retrofits [177]; (2) Assessment and information disclosure instruments are needed for benchmarking building energy consumption and identifying retrofit opportunities and operational issues [173]. These help to increase building owners’ awareness and encourage energy retrofits; (3) Research and service instruments are decisive for the development of state-of-the-art retrofit technologies and innovative retrofit plans to provide technological support for retrofit practitioners [176]; (4) State-funded financial incentive instruments are essential to support the enforcement of the previously mentioned instruments. As most retrofit activities require economic support, financial incentive instruments in the form of grants, rebates, preferential loans, or tax credits, are powerful tools that can reduce economic burdens on homeowners and encourage them to start retrofits [178]. At the same time, they can generate local economic markets and jobs [179]. Overall, government-administered financial incentive instruments must aim at subsidizing deeper retrofits, especially those that combine envelope thermal insulation, air tightness, and ventilation upgrades. The budget allocated for such instruments must conform to the need in improving the energy performance of existing buildings.

Reducing residential wood burning-related air pollution can be achieved by combining two main strategies. The need is (1) to reduce the energy demand through better building thermal performance, and (2) to transition from fossil fuel and wood burning equipment to efficient electrical systems for space and water heating, including the highly efficient ground- and air-source heat pumps and solar energy systems. Also, the electrification of households' energy systems would help to decrease indirect greenhouse gas emissions from electricity use in the longer term, as the Chilean grid electricity is expected to be generated 100% from renewable sources (i.e. solar, wind, and hydro) by 2040 [180]. In this sense, national programs such as the Air Quality Management Plans (PPDA) subsidizing building thermal reconditioning and wood stove replacement should be given continuity, particularly since they have proven to effectively reduce ambient PM_2.5_ in various Chilean cities, including Temuco where a reduction trend of 2.2 μg/m^3^ per year is observed [181]. Although the current programs were able to reduce PM_2.5_ emissions to a certain extent in highly polluted cities, much work remains to be done for PM_2.5_ concentrations to be within healthy levels. For instance, the eligibility for such PPDA grants needs to be extended to a greater share of households in urban areas, particularly the most vulnerable ones, as well as to households in rural areas presenting similar conditions of air pollution. Also, since the Ministry of Energy set ambitious national goals for the electrification of domestic energy systems [182], and new safety regulations for electrical system retrofits [183], it would be particularly appropriate to consider the implementation of a similar type of subsidy to foster the upgrade of outdated domestic electrical installations and prevent electrical failure hazard.

Another state subsiding mechanism is the Program for the Regeneration of Social Housing Complexes (i.e. in Spanish, Programa de Regeneración de Conjuntos Habitacionales, PRCH) included in the Supreme Decree Nº18 of 2017 [68], which seeks to improve the quality of life of families living in areas with a high degree of urban deterioration, where the access to services and public urban infrastructure is limited, and the housing thermal standards are poor [184]. The Program focuses on four dimensions: social, legal, housing, and urban, which allows for a holistic diagnosis and proposes solutions by the elaboration of a Master Plan designed through public participation (Ibid). Although the Program was created to respond to certain needs, budget constraints and normative problems have limited its widespread application to neighborhoods and cities. To that concern, there has been recent discussion among the public sector, expert professionals, and academics on improving the effectiveness of the PRCH in the context of the urgency for achieving better levels of sustainability in existing homes and buildings. Proposals for improving PRCH emphasize the importance of addressing critical issues, such as thermal and visual comfort, indoor air quality, and energy efficiency through housing retrofits, in association with other financing programs, to allow for more opportunities and better outcomes [185]. Increasing the associations of subsiding mechanisms from the Ministry of Housing and Urban Planning with policies and programs financed by other sectoral ministries, such as the Ministries of Energy, Environment, and Health, could allow for widespread improvements for homes in aged or deteriorated housing complexes. It bears noting that building energy retrofitting does not only help to reduce air pollution produced by the residential sector but also serves to improve the thermal comfort and health of building occupants while reducing greenhouse gas emissions at the country level.

### 4.2 Solutions for heating-related air pollution: improving thermal standards of new constructions

Long-term national policy actions are needed to reduce heating energy use in the residential sector. These actions must be viewed in an integrated way that includes the enforcement of regulatory codes for high-energy performance buildings, setting standards, technical requirements, and bans on envelope thermal insulation, air tightness, solar and internal gain management, ventilation, and energy systems, as well as renewable energy supply obligations. The new thermal regulation codes should aim at the net zero energy building (NZEB) performance level, and set maximum admissible values of primary energy consumption per unit of surface area for all new buildings, as well as obligations for retrofits. Regulatory codes should be complemented by a series of state funding instruments for new building constructions, as the implementation of new codes would affect production methods, construction habits, and costs. Like those described above, these instruments must encourage stakeholders to opt for improved construction practices and energy-efficient materials technologies. Specifically, grants, preferential loans, and tax incentives must be designed to favor thermal interventions targeting the NZEB level. Other financial instruments such as feed-in tariff schemes can encourage the production of electricity from renewable energy sources by requiring electric companies to purchase the electricity produced by renewable energy at a fixed tariff for a specified period.

In addition, efforts should be made to reduce the shortage of trained professionals across the industry and fill the technical and engineering skills gaps, by supporting the training of specialized workforces, as well as promoting the development of national industries able to manufacture energy-efficient materials, equipment, and technologies for the local market. Such measures can serve both the new construction and retrofit sectors in their adaptation to the new thermal regulation standards. Other initiatives, to be implemented either by governmental or non-governmental organizations, should focus on the creation of information campaigns to improve energy-saving awareness and help energy users to make efficiency-related choices. Moreover, there is a need for improving the relationship between energy consumers and suppliers to provide better data and make efficient technologies more affordable.

Regarding the challenge of inclusion of higher standards for the development of new housing units and buildings, in the meantime before the enactment of NZEB regulatory codes, another opportunity is the use of incentive mechanisms as defined by the Law on Impact Fees for Public Space [186]. Article 184 of this Law allows municipalities to offer developers the possibility to build projects with higher densities and construction coefficients or add more floors to residential and office buildings than originally allowed, in exchange for the construction of street-level public space, plazas, and/or green roofs. Note that Santiago is the only municipality that has set forth incentives for developers to consider the use of double-glazed windows for residential buildings, in exchange for special construction permissions, such as higher building height, density, and occupancy ratio than those defined in the Regulatory Communal Land Use Plans (PRC, for its acronym in Spanish) [187]. Since municipalities are responsible for designing their own PRC, new types of incentives aiming at improving thermal insulation and construction materials should also be created at the municipal level.

It is worth mentioning that the combination of all the above can have a significant impact on the building market and economic growth, aside from the benefits in the longer term on population wellness, environmental sustainability, and climate change resilience. Indeed, numerous new jobs can be created by the electrification of the national energy system [188] and by the implementation of governmental regulatory and financial incentives [179,189,190].

### 4.3 Solutions for water scarcity: implementing policies for water management

Established in 1981 under the dictatorship, the Water Code allows the state to grant perpetual water-use rights (DAA, for its acronym in Spanish) to individuals, which are entitled for managing the resource within private organizations. The law grants them the power to abstract water, distribute it among the right holders and carry out the necessary works for its use. However, the perpetual DAA constitute an issue in the context of water scarcity, because they represent more than six times the current national abstraction of fresh ground and/or surface water from natural sources [191]. Moreover, the DAA in 110 aquifers are greater than their recharge [191]. After a previous attempt to modify the Code to deal with the over-granted perpetual DAA, the new Water Code was finally approved in 2022. The Code now strengthens the statement that water should be considered a national asset for public use and recognizes its character of public interest for human consumption and sanitation, and the conservation of biodiversity [192]. It also provides more control to the state over the existing DAA. For the first time, the code sets grounds for the expiration of existing perpetual DAA in the case of non-use or non-registration, while allowing the state to grant new temporal DAA to users that best serve the public interest.

The World Resource Instituteś revealed that Chile is facing a high level of water stress [193]. Projections indicate that the situation will worsen in the future, as the ratio between total water withdrawals and available renewable surface water is expected to reach a value superior to 80% by 2040, and the country's water stress to be categorized extremely high, as a result of rising temperatures in critical regions and shifting precipitation patterns [194]. Trends illustrate that the level of 147 wells (72% of monitored wells) [191] and the level of streamflows in rivers [195] are decreasing. The main state agency designed to manage the country's water resources is the General Directorate of Waters (DGA, for its acronym in Spanish), which operates under the authority of the Ministry of Public Works. Based on rainfall, streamflow, and groundwater indices, the DGA is entitled to dictate water scarcity decrees, whose purpose, over their 1-year extendable duration, is to provide tools to minimize the damages derived from drought. Such water scarcity decrees empower the DGA to modify DAA by establishing limitations and/or additional permissions for water abstraction without the need to grant new DAA. The situation, as of July 2022, is that half of the country's communes have a water scarcity decree in force [196]. Copiapó has had eight water scarcity decrees between 2011 and 2022. Although these decrees seem justified as an emergency response, Palma [197] advised that they should be carefully regulated with technical support, for periods as short as possible, since the temporary modifications of DAA under these decrees result in less income for the companies that normally carry out maintenance works. Overall, there is a large consensus that the actual and projected levels of water stress require short- and long-term preventive measures, in which water scarcity decrees should not be the main policy for water management. The sustainable management of waters requires employing a combination of instruments able to induce reductions in water demand, improvements in use efficiency, reuse and recycling, and aquifer recharge. In the face of water scarcity, it is also relevant to review existing DAA which have not been adapted to the climate emergency. Populations and non-humans at risk (i.e. natural resources, ecosystems, and biodiversity) should have more legal protections against water abuse and malpractice. In this sense, DAA contracts must be granted only if they include enough safeguards and ensure repairs against spillovers, accounting first for the interests and prospects of populations and non-humans in any specific watershed.

To address water scarcity in the medium and long term, one of the national initiatives was the creation of the National Water Roundtable in 2019. This was a public-private instance where representatives of civil society, productive sectors, and executive and legislative branches were invited to seek joint medium- and long-term solutions. Its objectives were: (1) to establish the guidelines for a long-term water policy, (2) to define basic principles of a legal and institutional framework to support this policy, and (3) to propose necessary water infrastructure and water management practices at the basin level. This Roundtable generated a common diagnosis of the water situation, in which three challenges were identified: water security, protection of water quality and related ecosystems, and improvement of the legal and institutional framework, which were addressed in 12 thematic concepts [198]. As a result, the Government proposed various measures. First, a new bill submitted in June 2021 plans to establish a new institutional framework. This draft law intends to create the Secretary of Water Resources, a new authority responsible to dictate a National Policy on Water Resources, under the administration of the Ministry of Public Works and other organs such as ministerial commissions, national councils, and expert committees, in collaboration with different water stakeholders, including the DGA and water user organizations [199]. The aim is to promote the sustainable management of water and water security, providing guidelines, long-term strategy, and planning, favoring the use of water for human consumption, and integrated management of water resources. Secondly, an Emergency Plan was established in August 2021 to increase water availability and improve water use efficiency, so that water supply can be ensured for human consumption and food production [200]. The Plan contemplates increasing public inversions in desalination plant projects, dam constructions for irrigation of agricultural lands, infrastructure for drinking water supply to rural areas, as well as the implementation of taxation and sanctions to incentivize more efficient water use.

Water security, i.e. the provision of reliable and safe water in quantity and quality, where priority is given to human consumption, ecosystem conservation, and then the productive sector, is affected not only by climate change (i.e. more frequent droughts and flash floods), but also by the lack of adequate adaptation infrastructure, and the weak management due to the fragmentation of the institutions involved. New approaches proposed by the National Water Roundtable and the draft law include the incorporation of new water sources (including recycled water) and the development of new policy tools for integrated basin management. In the meantime, the DGA is organizing the introduction of new Strategic Water Management Plans (PEGH, for its acronym in Spanish) for different basins. These Plans involve the development of new works, actions, and managing schemes, based on surface and subsurface hydrological diagnosis, and considering sustainability, water quality, and factors affecting the security of supply. PEGH are projected to cover river basins of an area equivalent to 63% of the country in 2022 [201]. Yet, it is unclear whether the new PEGH will consider some solutions highlighted in the literature, e.g, the use of sustainable economic level of leakage [102], the determination of water tariffs that consider water scarcity [202], or the use of low energy-intensive technologies for wastewater treatment, such as constructed wetlands [203], microbial fuel cells [204], and anaerobic digestion and energy recovery from biogas combustion [205].

### 4.4 Solutions for soil pollution: implementing policies for soil management

Soil is considered a non-renewable natural resource that, in Chile, is threatened mainly by physical and pollution degradation due to human activities. Policies to promote soil conservation and protection are precarious or non-existent, therefore, it is urgent to incorporate soil into Chilean public policies, constituting together with water, a national asset for public use that must be protected and preserved for the conservation of ecosystems and the development of future generations.

Laws that regulate land resources in Chile are widely dispersed through different programs, plans, and committees belonging to different organizations [206]. The fragmented public policies represent a weakness for the sustainable management of natural resources in the country, as the authorities related to soil issues (i.e. the Ministries of Mining, Environment, Agriculture, and Housing and Urban Planning) have distinct services, and the monitoring information of soil resources does not benefit from central coordination at the national level. Besides, the committees in charge of evaluating environmental impact assessments of soil exploitation activities are formed by ministers designated by the President of the Republic. This situation has led Chile to base a substantial part of its economy on the extraction of natural resources, prioritizing economic interest over sustainability questions. For example, mining activities involving metal enrichment were allowed to operate despite producing toxic residues [75], as Chile does not have regulations for maximum permissible concentrations of metals in soils [79].

The Chilean land-use and urban legislation are vague regarding the consideration of hazardous areas. So, there is a need to create a policy framework that can classify exposed and contaminated soils as risk areas. Specifically, discussions are ongoing since 2019 for the formulation of a soil framework law proposing the creation of a national soil institute for the generation of new information about Chilean soil properties [82,207]. According to Swartjes et al. [208], there should be four aspects to consider in a contaminated site management policy framework: a pollution mitigation strategy, relevant soil quality standards, the urgency of actions, and sustainable soil management. Moreover, Li et al. [209] propose a flow of technical information and a decision-making diagram for soil remediation projects. To achieve action remediation in Chile, there are a few important steps that remain to be implemented: (1) the development of a proper legislation framework that considers sustainable practices, (2) the identification of knowledge gaps on soils, and (3) the setting of soil quality standards. Moreover, it is particularly important to proceed with soil standards, based on the critical threshold of pollution established by authorities, to implement updated laws and decrees that meet actual sustainable development goals. This should also include urban soils, which are affected by pollution from urban activities, particularly the accumulation of polluting materials [70,75,210].

## 5 Conclusions: toward sustainability and resilience in Chile and elsewhere

We summarized recent accomplishments and gaps in the path Chile has followed toward urban sustainability and resilience, mostly from an environmental perspective, on issues associated with PM-related air pollution from the residential sector, the urban water cycle, and soil contamination. Due to its extremely diverse geography, extending from 17° South to 56° South, the country must be prepared to deal with various types of situations and issues. The focus has been on the bonds between the geophysical context and the Chilean cities, which are prone to both short-term (e.g. floods, earthquakes) and long-term (e.g. pollution, water scarcity) threats to ecosystem health and human wellness. The Temuco and Copiapó case studies highlight how tackling these challenges requires solutions and actions on multiple scales and areas, including assessment and information, regulation and financial incentive measures, and infrastructure and technologies. For the development of a more sustainable and resilient urban environment, Chilean cities must address the following issue that may guide the transitional paths of many other cities elsewhere.

a. **The development of public policies aiming at reducing air, water and soil pollution**: examples of such policies include (1) upgrading environmental standards limiting nutrient concentrations in water bodies, particularly to reduce and eventually eliminate the discharge of nitrogen and phosphorus to the environment, (2) implementing stricter building energy codes for new constructions, as well as for the retrofit of existing buildings, to improve building thermal performance, reduce space heating energy use, and lower PM and greenhouse gas emissions, (3) creating information campaigns to improve energy saving awareness and help energy users to make efficiency-related choices and (4) implementing region-wide financial incentives targeting homeowners in both urban and rural areas, to boost building energy retrofit interventions, especially those deeper refurbishments that combine envelope thermal insulation, air tightness and ventilation upgrades, and (5) supporting the development of local industries and the training of specialized professionals.
b. **The improvement of water infrastructure and technologies**: there are several opportunities to improve, replace or implement technological solutions. Some examples of this are (1) the maintenance of current infrastructures, such as the retrofit of drinking water distribution systems to reduce leakages, (2) the replacement of older technologies by more efficient and complete ones (e.g. the replacement of marine outfalls with adequate wastewater treatment technologies), and (3) the implementation of newer or alternative technologies, such as sustainable urban drainage and greywater reuse systems to increase the conservation and availability of water resources, as well as renewable energy systems to produce clean and sustainable energy for water treatment facilities.
c. **The implementation of new risk management systems**: examples of these systems include (1) environmental and health monitoring systems able to provide early warnings and information about potential risks (e.g. early warning of high turbidity events in rivers), and (2) management systems considering the energy-water nexus in the use of critical resources.
d. **The implementation of strategies to reduce vulnerability to natural hazards**: new actions and infrastructure are needed to decrease levels of risk. Examples include the Kaukari Park which limits the exposure to mudslides in Copiapó, or the Agenda 2030 developed by the water regulator to prevent or minimize the impact of natural hazards on the urban water systems.
e. **The consideration of risks from an anthropogenic origin in urban planning**: considering a variety of risks in urban planning requires changes in legislation. For the Chilean case, such changes should affect the General Law of Urban Planning and Construction (OGUC), which establishes the types of risks to be considered when limiting urban development in planning instruments. Furthermore, new guidelines must be developed to provide a framework for the quantitative assessment of potential risks and exposed areas. An example of this is the soil quality standards, which are key to limiting urban development in areas near mine tailings.

Our recent experience shows that scientific research is key to providing the basis for effective decision-making in urban environments. We believe this science-public policy alliance must be strengthened further to address the present need for the conservation of ecosystems and prevent future vulnerabilities to hazardous situations and climate change. Examples of current research goals in Chile are: (1) the identification of sources and the understanding of pollutants dynamics in aquatic environments; (2) the understanding and modeling of factors triggering high-turbidity events in rivers used as drinking water sources; (3) the development of novel technologies for the treatment and recycling of wastewater; and (4) the development of technologies facilitating efficient use of energy and water at industrial and residential scales within a regulatory framework that ensures their massive implementation.

## Author contribution statement

François Simon, Jorge Gironás, Javier Rivera, Alejandra Vega, and Guillermo Arce: Conceived and designed the experiments; Analyzed and interpreted the data; Contributed reagents, materials, analysis tools or data; Wrote the paper.

María Molinos-Senante, Héctor Jorquera, Gilles Flamant, Waldo Bustamante, Margarita Greene, Ignacio Vargas, Francisco Suárez, Pablo Pastén, and Sandra Cortés: Analyzed and interpreted the data; Contributed reagents, materials, analysis tools or data; Wrote the paper.

## Data availability statement

Data included in article/supp. material/referenced in article.

## Declaration of competing interest

The authors declare that they have no known competing financial interests or personal relationships that could have appeared to influence the work reported in this paper.
